# A gene–culture co-evolutionary perspective on the puzzle of human twinship

**DOI:** 10.1017/ehs.2024.30

**Published:** 2024-11-11

**Authors:** Augusto Dalla Ragione, Cody T. Ross, Daniel Redhead

**Affiliations:** 1 Department of Human Behavior, Ecology and Culture, Max Planck Institute for Evolutionary Anthropology, Leipzig, Germany; 2 Department of Sociology, University of Groningen, Grote Rozenstraat 31, 9712 TG Groningen, The Netherlands; 3 Inter-University Center for Social Science Theory and Methodology, University of Groningen, Groningen, The Netherlands

**Keywords:** Twinship, coevolution, twins, geminophilia, geminophobia

## Abstract

Natural selection should favour litter sizes that optimise trade-offs between brood-size and offspring viability. Across the primate order, the modal litter size is one, suggesting a deep history of selection favouring minimal litters in primates. Humans, however – despite having the longest juvenile period and slowest life-history of all primates – still produce twin births at appreciable rates, even though such births are costly. This presents an evolutionary puzzle. Why is twinning still expressed in humans despite its cost? More puzzling still is the discordance between the principal explanations for human twinning and extant empirical data. Such explanations propose that twinning is regulated by phenotypic plasticity in polyovulation, permitting the production of larger sib sets if and when resources are abundant. However, comparative data suggest that twinning rates are actually highest in poorer economies and lowest in richer, more developed economies. We propose that a historical dynamic of gene–culture co-evolution might better explain this geographic patterning. Our explanation distinguishes *geminophilous* and *geminophobic* cultural contexts, as those celebrating twins (e.g. through material support) and those hostile to twins (e.g. through sanction of twin-infanticide). *Geminophilous* institutions, in particular, may buffer the fitness cost associated with twinning, potentially reducing selection pressures against polyovulation. We conclude by synthesising a mathematical and empirical research programme that might test our ideas.

## Graphical abstract

**Figure:**
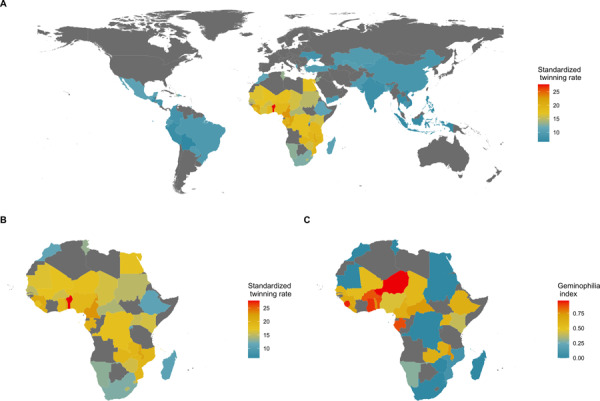

**Social media summary:** Why do twinning rates vary between human groups? We suggest that gene–culture co-evolution might play a role.

Twins saw the houses of great personages but did not go there […]
Instead they entered the houses of the poor […]
They made the poor rich […]
With reputation of wealth and fecundity. (Traditional Yoruba *oriki*; Oruene, 1985)

## Introduction

Since the pioneering work of Lack (1947) on clutch size in birds, life history theorists have proposed that natural selection should favour animal litter sizes that solve trade-offs between offspring quantity (i.e. brood size) and offspring viability and/or fecundity (i.e. offspring quality). As a result, modal litter sizes will typically be lower than what is biologically possible. Empirically observed clutch/litter sizes in natural populations, however, are usually even smaller than predicted optimal values (Godfray et al., 1991). This is perhaps evidence of further trade-offs between current and future reproduction (Godfray et al., 1991; Sikes & Ylönen, 1998).

Within the mammalian class, there is substantial variation in litter size – with values ranging from as large as 32 in the genus *Tenrec* (Olson, 2013) – a reproductive pattern called *polytoky*, to values as small as one in chimpanzees, humans and several other primate species (Leutenegger, 1979) – a reproductive pattern called *monotoky*. Researchers regard polytoky as the ancestral state of extant mammalian monotocous species, with monotoky being an evolutionary novel trait that several mammalian species evolved (Lukas and Clutton-Brock, 2020; Leutenegger, 1979; Garbino et al., 2021). The widespread monotoky observed among most primates appears to be associated with a suite of other life history characteristics, including extended periods of juvenile development and long lifespans (reviewed in: Jones, 2011).

Although most primates are monotocous, several species, including humans, have maintained the propensity to give birth to multiple simultaneous offspring (i.e. ‘twins’) at relatively low, but appreciable, frequencies (Geissmann, 1990). One type of twinning is monozygotic twinning, which results from the fertilisation of a single ovum that undergoes a process of splitting. Monozygotic (or ‘identical’) twins therefore share the same genome and are always of the same sex. The majority of twin births, however, result from dizygotic twinning (i.e. they result from the fertilisation of two separate ova by two different sperm). This entails that the twins share half of their genome, like non-twin siblings.

Monozygotic (MZ) twinning is normally thought to result from random biological processes, and it occurs at a low, geographically invariant rate across human populations (Bulmer, 1970; Hoekstra et al., 2008b); MZ twinning in humans is not all that puzzling. In contrast, dizygotic (DZ) twinning results from polyovulation (i.e. the release of multiple ova during a single fertility cycle), and shows signs of both genetic heritability (Hoekstra et al., 2008b; Duffy & Martin, 2022) and geographic heterogeneity, occurring at variable rates in human populations (e.g. from 0.7% to 2.7% of all births; Rickard et al., 2022). Dizygotic twinning in humans is puzzling, both for its persistence despite apparent costs, and for its relatively large geographic variation. Estimates of twinning rates in other primates are scarce and are probably unreliable owing to small sample sizes (Geissmann, 1990).

A body of empirical work suggests that twinning among monotocous species usually entails significant biological costs for both mothers (e.g. in terms of higher risk of maternal mortality; Senat et al., 1998) and offspring (e.g. in terms of lower birth weights and higher risk of infant mortality; Monden & Smits, 2017). In the absence of mechanisms to counteract these costs, it seems unlikely – at first glance – that natural selection would have maintained a propensity for DZ twinning (Anderson, 1990).

The leading candidate explanation for the persistence of DZ twinning in humans links DZ twinning to ecological conditions (Lummaa et al., 1998). The argument states that plasticity in polyovulation (and thus DZ twinning) may be fitness enhancing, even among modally monotocous species, if it leads to recruitment of larger sib sets in ecologies where resources are abundant and the costs of twinning are lower. However, this explanation, while setting the groundwork for a phenotypic plasticity perspective on twin births, conflicts with comparative data suggesting that the incidence of DZ twinning is actually highest in West and Central Africa (Smits & Monden, 2011), regions characterised by substantial resource insecurity relative to more developed economies, where the twinning rate is lower.

To resolve the puzzle of human twinship, we propose a gene–culture co-evolutionary process that builds upon the notion that polyovulation may be a phenotypically plastic response to ecological conditions, but integrates the idea that cultural institutions can be an essential component of the environment to which such responses are adapted. We introduce the idea of *geminophilous* and *geminophobic* cultural institutions as those that – respectively – celebrate and materially support twins (e.g. through third-party provisioning of twins, and/or conferral of prestige on twins or their parents) and those that malign and repudiate twins (e.g. through sanction of twin infanticide or conferral of contempt on twins or their parents). Such systems may lead to significant survival and reproductive consequences for individuals expressing the DZ twinning phenotype, potentially operating as selective forces on the genes involved in regulation of polyovulation. We argue that such cultural institutions might be sufficiently strong, and that population-level variation in them might be sufficiently large, to explain population-level variance in the incidence of DZ twinning. If our explanation is correct, it would entail several empirically testable predictions, which could be evaluated both through ethnographically informed quantitative research and through genetic research.

In what follows, we provide a brief overview of the literature describing the etiology and geographic distribution of DZ twinning, and link this literature to evolutionary thinking on DZ twinning in humans. We then introduce readers to the idea that it is not just variation in DZ *twinning* (a biological phenomenon), but also variation in *twinship* (a cultural phenomenon) that requires an evolutionary explanation. Note that we use the word *twinship* here to refer to the beliefs, practices and cultural institutions that govern how twins should be treated. We then provide readers with the ethnographic context needed to appreciate the remarkable breadth of variation in twinship systems cross-culturally. Finally, by integrating ideas from the field of cultural evolution, we synthesise a gene–culture co-evolutionary model of DZ twinning. We conclude by outlining an empirical research programme that would test our ideas.

## The etiology and geography of twinning

Monozygotic twinning is thought to be an essentially random event, with its incidence in humans being constant across space and time (Bulmer, 1970). Dizygotic twinning is the most prevalent type of twinning and it does not occur randomly – i.e. it is associated with a diverse set of explanatory factors, from ecological/behavioural variables to genetic ones (Hall, 2003). Finally, a third type, sesquizygotic twinning, has been identified in recent years (although non-MZ and non-DZ types of twinning have long been theorised; e.g. Bulmer, 1970). The offspring of sesquizygotic pregnancies share a proportion of genes that is intermediate between dizygotic and monozygotic twins (Gabbett et al., 2019). Since we are particularly interested in the population-level distribution of DZ twinning rates, this article will focus on this type of twinning.

The health costs of twinning for both mother and offspring in humans are well described. Such costs include: increased risk of maternal death (Senat et al., 1998), congenital anomalies (Hall, 2003) and low birth weight, resulting in disproportionately high perinatal, neonatal and infant mortality (Elster et al., 2000). In a case study from rural Tanzania, Minocher et al. (2023) use data from a 20-year prospective study to show that twins have a 35% chance of death before age 5, in comparison with singletons, who have a 21% chance of death over the same interval. Similar costs have been observed in both developing (Monden & Smits, 2017; Vogel et al., 2013) and developed economies (Smith et al., 2014; Kleinman et al., 1991; Monden & Smits, 2017). Both MZ and DZ twins have higher risks of low birth weight and congenital anomalies compared with singletons (although MZ twins more so than DZ twins; Hall, 2003). There may be reason to think that twinning is similarly costly for other primates as well, as there are similar trade-offs related to parental provisioning and offspring development (Link et al., 2006; Chapman &Chapman, 1986). Twinning is also likely to affect parents’ future reproduction, owing to its direct (e.g. in terms of mortality risk) and indirect (e.g. in terms of parental investment costs) effects on mothers. Indeed, an analysis of several pre-industrial European populations concluded that a twinning event decreased the chance of a future birth, ultimately leading women with higher twinning propensity to have lower reproductive output (Rickard et al., 2022).

### Individual and ecological factors

Dizygotic twinning is variably associated with a host of ecological, behavioural and physiological risk factors. In line with the idea of polyovulation being a plastic, state-dependent adaptive strategy – whereby individuals with robust phenotypes may benefit from ‘doubling up on reproductive rate’ when conditions are good – maternal anthropometrics – such as body mass index (Basso et al., 2004; Reddy et al., 2005; Hoekstra et al., 2010) and height (Hoekstra et al., 2010; Bortolus et al., 1999) – have been found to be positively associated with increased likelihood of DZ twinning. Alongside this, older women appear more likely to conceive twins than younger women (Beemsterboer et al., 2006; Ananth & Chauhan, 2012), possibly as the result of an evolved, age-dependent polyovulation strategy designed to offset the higher embryo mortality risks occurring at later ages (Hazel et al., 2020). The higher twinning rate of older women has also been suggested to represent ‘terminal reproductive investment’ (Helle, 2008).

Correlations have been found between DZ twinning rates and a range of other variables. Parity, for example, has been found to be positively associated with twinning risk, independent of maternal age (Bulmer, 1970). Other studies have found positive associations between smoking and DZ twinning propensity (Hoekstra et al., 2008b, 2010; Källén, 1998). Similarly, as we will see below, some researchers have attributed the high rates of twinning in West Africa to dietary habits (Steinman, 2006a). More specifically, researchers have found that certain species of yam produce estrogen-like compounds that might increase polyovulation rates in humans, leading to a surfeit of twin pregnancies where consumption of such yams is common (Nylander, 1979; Marinho et al., 1986; Steinman, 2006a,b). Finally, medically assisted reproduction has been shown to increase the likelihood of producing twins (Hoekstra et al., 2008b), but the causal mechanism here is not at all ambiguous. In fact, the sharp rise in twinning rates in developed countries in the past few decades has been driven mostly by the widespread use of fertility treatments, which limits the usefulness of twinning data from developed countries in comparative studies aiming to understand ‘natural’ variation in twinning rates. However, increased maternal age does appear to be an important secondary factor in the recent increase in twinning rates in developed economies (Pison et al., 2015; Ananth & Chauhan, 2012).

Researchers have also attempted to leverage temporal variation in twinning rate (holding constant the population of interest) in order to test how resource shocks or other changes in ecological circumstances (e.g. famines or wars) affect twinning risk. For example, a stark decline in twinning rate was documented in Tokyo (Nakamura et al., 1990) and in several European countries during World War II (Bulmer, 1959), with the fluctuations being driven by DZ twinning rates, while MZ twinning rates remained unperturbed. It has therefore been posited that poor maternal state decreases the chance of polyovulation, a necessary condition for DZ twinning (Bulmer, 1970). Since MZ twinning rates remained constant, even during such periods of material deprivation, plummeting DZ twinning rates are better explained by ovulatory changes, rather than changes in embryo or foetal mortality (which would arguably have affected MZ twinning rates as well). However, in Scandinavia, twinning rates did not appear to vary with catastrophic events, such as wars or famines (Eriksson et al., 1988). Furthermore, several European countries actually experienced declines in twinning rates only years after World War II (starting around the late 1950s), or even way before it (e.g. France's twinning rate started to decline after World War I and was unaffected by World War II; Pison and d'Addato, 2006). This heterogeneity casts doubt on the generalisability of conclusions from studies viewing wartime conditions as particularly salient drivers of variation in twinning rate.

### Geographic distribution

**Figure 1.**
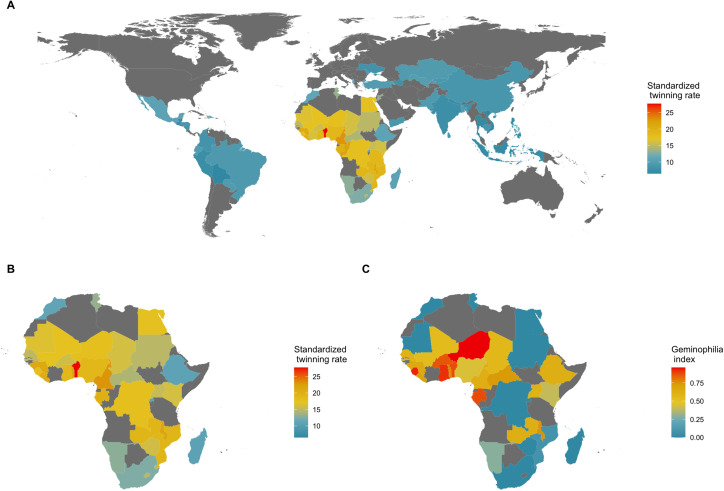
Geography of twinning rate and norms about the treatment of twins. (a) National twinning rate per 1000 births (adjusted for average maternal age) in 76 countries; data from Smits and Monden (2011). (b) A closer look at Africa. (c) Percentage of land area historically held by predominantly non-twin-killing groups, a proxy for geminophilous norms; data from Fenske and Wang (2023, fig. 4), who retrieved the information from Murdock (1959). Naive country-level regressions suggest that there are 3.84 (95% confidence interval, CI, 0.72, 6.96; adjusted *R*^2^ = 0.12; *N* = 36; *P* = 0.017) more twin births *per mille* in countries where non-twin-killing groups make up the entire population, relative to countries where such geminophilous norms are not documented. Such an analysis, however, treats an absence of evidence of geminophilous norms, as evidence of absence. Restricting the sample to countries for which at least 50% of territory is unambiguously coded as historically populated by either twin-killing or non-twin-killing groups, the coefficient increases to 4.17, but the confidence region expands (95% CI, −3.02, 11.36; adjusted *R*^2^ = 0.02; *N* = 21; *P* = 0.241) to include the value of 0, owing to the smaller sample of countries. Finer-scale models are needed to make such comparative analyses rigorous, as simple regressions are subject to ecological confounding.

Stark between-population differences in rates of DZ twinning are observed at both global and regional scales (Hoekstra et al., 2008b). The highest rates of DZ twinning are found in the Western and Central regions of Sub-Saharan Africa, and the lowest rates are found in the Southern and Eastern regions of Asia (Smits and Monden, 2011; Hoekstra et al., 2008b). In particular, the West African country of Benin has been reported to have the highest twinning rate in the world, with a twinning rate of 2.7% – which is roughly four times larger than that of most Asian countries (Smits & Monden, 2011). Additionally, the Yoruba ethnic group – which resides mostly in the neighboring country of Nigeria – has long been the focus of studies on twinning owing to the high frequency of twin births occurring in that sub-population (Creinin & Keith, 1989; Nylander, 1970, 1979). A high-twinning regional cluster, therefore, appears to exist in West Africa (see Fig. 1).

Extant research has favoured ecological accounts of such geographic variation, rather than genetic ones, pointing to evidence of changes in DZ twinning incidence as a function of variation in environmental risk factors (Nylander, 1970), especially diet (Marinho et al., 1986). This idea is supported by some independent lines of evidence; for example, some work suggests that different ethnic groups (i.e. Euro-descendents and Afro-descendents) living in the same area in Costa Rica have similar twinning rates (Madrigal et al., 2001), and other work has shown that immigrants’ twinning rates tend to diverge from those of their countries of origin (Pollard, 1995). However, the evidence here is mixed. In the USA, for example, although group-level differences in twinning rate between immigrant ethnic groups may have weakened, they still persist (Pollard, 1995; Khoury & Erickson, 1983; Abel & Kruger, 2012), with the highest rates of twinning being found among Americans of African ancestry. Likewise, evidence from the Demographic and Health Survey data in developing countries, shows that Haiti – a population of mostly West African ancestry – has a markedly higher twinning rate than other countries in the wider Latin American region (Smits & Monden, 2011).

Although it is plausible that diet explains part of the variation in twinning risk, it is just as plausible that genetic differences do contribute to it. The strongest evidence in favour of a genetic component to twinning rate in humans comes from the existence of small, regional population clusters with exceptionally high twining rates. For example, a genetic founder effect appears to explain the high twinning rate (of 2%) observed in the small Brazilian town of Cândido Godói, which is inhabited by a homogeneous population of German descent (Tagliani-Ribeiro et al., 2011). Similar high-twinning-rate clusters – probably genetic in origin – can be found throughout Europe (for example, in the French regions of Brittany and Massif Central; Bulmer, 1959). Looking beyond human data, the genetic underpinnings of polyovulation and twinning have been extensively studied in non-human mammals.

### Genetic factors

Owing to obvious economic incentives, farm animals have been the subject of considerable research on the genetic basis of twinning (e.g. in cattle, a monotocous species) and litter size (e.g. in sheep, a frequently polytocous one). Studies have demonstrated that ovulation rates show strong signs of genetic heritability in both cattle and sheep (Vinet et al., 2012). Polyovulation is considered to be a quantitative trait in these animals, because the release of multiple eggs per estrous cycle can be achieved through several different and non-exclusive biological processes (Vinet et al., 2012). There is also strong evidence of phenotypic plasticity in ovulation: several breeds of sheep alter their ovulation rate based on body condition (Martin et al., 2004). Although ovulation rate is highly heritable in cattle, twinning rate is less heritable than ovulation rate, since twinning is dependent on both polyovulation and on environmentally influenced processes that act as filters, including fertilisation and embryo/foetal mortality (Vinet et al., 2012). It is possible that fitness payoffs to different rates of ovulation may vary depending on environmental/maternal conditions, with consequences in terms of the evolution of plasticity and population-level differences in ovulation and twinning, an idea we explore with a simple formal model in Box 1.

Box 1: Selection on ovulation strategies

Here, we present a simple model to illustrate how the relative fitness of different ovulation strategies may vary depending on environmental conditions, and how phenotypic plasticity may have been selected for as a consequence. For simplicity, let us assume the existence of two competing genotypes – a double-ovulation genotype, *P*, and a mono-ovulation genotype, *S*, with fitnesses, *W*_*P*_ and *W*_*S*_, respectively. Individuals ovulate, and the ovum is fertilised. The ovum then goes through a phase of embryo/foetal mortality selection. Let us assume that mortality, *m*, is a decreasing linear function of an individual's resource endowment, *E* ∈ (0, 1). Then:
1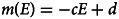 with *c* > 0 and *d* > *c*.

Next, let us assume that the reproductive value of a singleton birth is a constant, *R*_*s*_, and that the reproductive value of a twin birth is a linear function of resource availability:
2
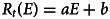

We assume no trade-offs between current and future reproduction, and no trade-offs in resource allocation between embryo/foetal mortality reduction and postnatal investment. Then, we construct fitness expressions for *P* and *S*:
3
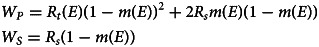

The fitness of the double-ovulation genotype, *P*, is given by the reproductive value of a twin birth, *R*_*t*_(*E*), times the probability of producing twins, (1 − *m*(*E*))^2^, plus the reproductive value of a singleton birth, *R*_*s*_, times the probability of producing a singleton, 2*m*(*E*)(1 − *m*(*E*)). The fitness for the mono-ovulation genotype, *S*, is similarly constructed.

Twinning is costly – i.e. it yields a lower fitness payoff than a singleton birth, *R*_*t*_(*E*) < *R*_*s*_ – in the interval [0, E*]. Above the resource threshold, E* = (*R*_*s*_ − *b*)/*a*, twinning is adaptive regardless of embryo/foetal mortality levels, and natural selection favours genotypes which maximise twinning, leading to polytoky. Below E* (i.e. within the costly twinning interval), the fitness of *P* is higher than *S* when the following inequality is satisfied:4
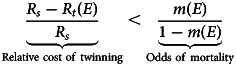

**Figure 2.**
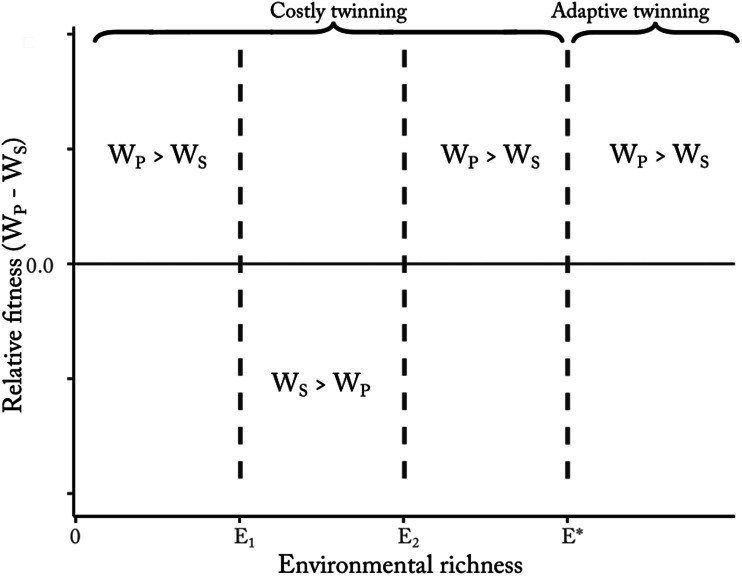
Relative fitness of double-ovulation as a function of resource endowment. *W_P_* is the fitness of the double-ovulation genotype; *W_S_* is the fitness of the single-ovulation genotype. Note that the *x*-axis is not necessarily partitioned in evenly spaced intervals; this is done here just for visualisation purposes.

Gathering the terms of the inequality above yields:5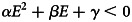 where *α* = −*ac*, *β* = *a*(*d* − 1) + *c*(2*R*_*s*_ − *b*), and *γ* = *R*_*s*_(1 − 2*d*) + *b*(*d* − 1). The expression on the left-hand side is a concave parabola (the quadratic term is negative). Let us assume that the two roots {*E*_1_, *E*_2_} of the parabola lie within the interval [0, E*], so that the inequality is satisfied for 0 < *E* < *E*_1_ and *E*_2_ < *E* < E*. The resulting resource space is then partitioned into four different regions defined by the adaptiveness of twinning and the adaptiveness of double-ovulation, as shown in Fig. 2. For resource levels lower than E*, twinning is maladaptive, but double ovulation may nonetheless be selected for. Such a fourfold partition is not the only possible modelling outcome – the plane can be partitioned into a smaller number of regions, depending on the location of the roots of the parabola in Eqn (5). Nevertheless, the model illustrates how the relationship between the fitness payoff of double ovulation and environmental/resource condition may be non-monotonic. It may be optimal to double-ovulate at both low and high resource levels, even when twinning is costly – e.g. for *E* < E*. Such non-monotonicity reflects the interplay between the costs of twinning and the risk of embryo/foetal mortality.

A number of implications follow. Populations inhabiting environments with stable levels of resource availability will be selected towards one ovulation strategy or the other. In a fluctuating environment, however, a genotype which regulates ovulation rate based on environmental cues could be favoured by selection, since it would be able to adaptively adjust as resource conditions change. Such mechanisms appear to be found in mammals – for example, there is evidence of ovulation rate being a phenotypically plastic trait, controlled by body condition, in several breeds of sheep (Martin et al., 2004). Additionally, and counterintuitively, we might expect to see high twinning incidence in particularly bad environments where the twinning phenotype is markedly maladaptive, if its relative costs are lower than the pressure exerted by embryo/foetal mortality, as outlined in Eqn (4). Finally, a subtle implication of the model for empirical studies is that we are unlikely to be able to infer selection gradients on polyovulation based on inferences drawn from comparisons between twinners and non-twinners. This is because twinners are only one subset of the population carrying the the ‘double-ovulation’ genotype, and their reproductive success does not necessarily represent the genotype's fitness. Since fitness is a population-level quantity, a double-ovulation genotype might still be selected for even if twin-producing individuals bearing the genotype attain lower reproductive success because of the cost of twinning.

As observed in other mammals, DZ twinning in humans appears to be a complex trait influenced by multiple genes (Painter et al., 2010), which nevertheless shows signs of genetic heritability (Hoekstra et al., 2008b). Women with a familial history of DZ twinning have a higher risk of having DZ twins themselves (Hoekstra et al., 2008a; Meulemans et al., 1996; Lewis et al., 1996). Multi-generational pedigree data from several European, Euro-descendent, and West African populations suggest heritabilities (*h*^2^) of twinning in the 8–20% range (Duffy & Martin, 2022). These values are likely to be underestimates of the true heritability of DZ twinning, however, because the authors did not differentiate between DZ and MZ twinning. It is of note that estimates of *h*^2^ for twinning in the West African samples of Duffy and Martin (2022) are similar to the estimates for European and Euro-descendent populations (see also Hur et al., 2024), problematising previous claims that twinning probability in West Africa did not vary by lineage (Nylander, 1970), which implied that phenotypic variation was solely attributable to environmental factors, such as diet.

Paralleling study designs used previously in cattle and sheep, researchers have tried to identify candidate genes – e.g. follicle-stimulating hormone and growth-differentiation factor-9 – associated with DZ twinning rate in humans (Beck et al., 2021). Although the role of genetics in the propensity for DZ twinning in mammals has been known for a long time (White & Wyshak, 1964), evidence of its specific genetic underpinnings, especially in humans, has only started emerging with the rise of genome-wide association studies. Recent genome-wide association study protocols have identified several new loci associated with the outcome of DZ twin production (e.g. FSHB, FSHR, SMAD3, GNRH1, ZFPM1 and IPO8), and population-level polygenic risk scores have been found to predict twinning rate at the country level, especially in Africa (Mbarek et al., 2024). Additionally, Mbarek et al. (2024) found signatures of past selection against some alleles associated with DZ twinning. Since the study of the genetics of DZ twinning is still an active research programme, we will reason about its evolutionary dynamics using models which make the ‘phenotypic gambit’ (Grafen, 1991), and thus treat a complex trait like polyovulation rate *as if* it were controlled by a single locus.

## Evolutionary significance

The evolutionary literature on human twinning typically approaches the problem in one of two ways: some work builds on the notion that twinning is itself a maladaptive by-product of the evolved propensity to polyovulate (the ‘*insurance-ova hypothesis*’; Anderson, 1990), and other work advances the idea that twinning is an expression of an underlying high-fecundity phenotype (the ‘*phenotypic quality hypothesis*’; Hoekstra et al., 2008b; Robson & Smith, 2011). In Box 1, we present a simple formal model for the relative fitness of polyovulation that recovers both of these hypotheses as special cases.

According to the insurance-ova hypothesis, DZ twinning is a by-product of polyovulation. Polyovulation is argued to offset the risk of embryo mortality, increasing the chances that at least least one fertilised egg can be brought to term (Anderson, 1990). In a subset of pregnancies, however, more than one fertilised egg may survive, resulting in a twin birth. Since twinning is associated with a host of mortality and morbidity problems, both for mothers and for offspring themselves (Monden & Smits, 2017; Conde-Agudelo et al., 2000; Ghai & Vidyasagar, 1988), the hypothesis regards the multiple-births outcome as a relatively rare, maladaptive collateral trait. Formal models that build on the insurance ova hypothesis highlight how an age-dependent polyovulation mechanism might evolve to account for increasing embryo mortality in older women, which would explain why maternal age is a risk factor for DZ twinning (Hazel et al., 2020).

On the other hand, according to the ‘phenotypic quality’ hypothesis, DZ twinning is an adaptive response driven by underlying phenotypic quality – e.g. sufficiently high BMI (Sear et al., 2001; Lummaa et al., 1998). This hypothesis predicts that twinning will be associated with other fitness-relevant life history traits (such as generally high fecundity). Several studies on the fitness consequences of twinning show that mothers of twins tend to have higher reproductive success than non-twinners (operationalised in diverse ways), and conclude that twinning propensity might be under selection as an expression of an advantageous latent ‘intrinsic fertility’ phenotype (Lummaa et al., 1998; Sear et al., 2001; Helle et al., 2004; Gabler & Voland, 1994; Robson & Smith, 2011; but see Haukioja et al., 1989 and Rickard et al., 2022). In support of this idea, the relative reproductive success of DZ twinners in pre-industrial Finland was observed to vary by region, with DZ twinning mothers attaining higher lifetime reproductive output than non-twinning mothers in areas with constant and abundant resources, but lower lifetime reproductive output in poorer/more variable environments (Lummaa et al., 1998). Recent research, however, has cast some doubt on these conclusions, as the analytical strategies used to evaluate the empirical data may have failed to account for differential exposure to the total risk of twinning (see Rickard et al., 2012, for technical details).

The insurance ova hypothesis and the phenotypic quality hypothesis appear to make different predictions about when DZ twining rates should be high. The insurance ova hypothesis leads us to expect higher polyovulation rates when conditions are bad, and the risk of embryo mortality high, while the phenotypic quality hypothesis leads us to expect higher polyovulation rates when conditions are good, and the potential cost to rearing twins low. Upon deeper inspection, however, the insurance ova hypothesis and the phenotypic quality hypothesis are actually special cases of a single unified model.

In Box 1, we outline a simple ecological model of optimal ovulation strategy as a function of resource availability. This unified model shows that polyovulation can be adaptive at both low and high extremes of environmental richness. An implication of the model is that a genotype which flexibly regulates ovulation based on environmental cues might be favoured by selection, potentially explaining variability in ovulation propensities across different environments. Such a model serves to explain both why DZ twining appears linked with individuals of robust phenotype (Sear et al., 2001), and why the highest DZ twinning rates are found in developing countries, where undernutrition is common (ESHRE Capri Workshop Group, 2006).

Until now, we have considered ecological effects on twinning to reflect simple impacts of the natural environment, omitting causal scope for cultural institutions to influence twinning propensity. In the remainder of the paper, we hope to demonstrate that cultural institutions related to twinning are an essential component of the environment to which polyovulation rates must be adapted. Moreover, we argue that cultural institutions related to twinning and genetic/phenotypic variation in polyovulation may be deeply interdependent.

## Twinship and cultural systems

Twinship is a recurrent element of cultural institutions related to fertility around the world (Leroy, 1976; Renne & Bastian, 2001). Such cultural institutions govern how twins should be treated, both materially and socially. The range of attitudes and behavioural responses that twins evoke is diverse – ranging from twins being viewed as legitimate targets of infanticide (Granzberg, 1973) to twins being celebrated as supernatural sources of wealth and good luck (Herskovits, 1938; Saulnier, 2009). We refer to the cultural institutions surrounding twinship as *geminophobic* or *geminophilous*, depending on whether they treat twins with contempt or celebration, respectively. Ample ethnographic evidence suggests that Sub-Saharan Africa, in particular, is rife with various norms and beliefs related to twinship (Pison, 1987; Leroy, 1976), and we argue that this variation in cultural norms is just as worthy of investigation as variation in twinning rate itself.

Negative twinship salience (i.e. geminophobia) can have detectable demographic consequences. For example, there is evidence that twins suffered disproportionately high infant and child mortality rates in African societies where the practice of twin infanticide was socially sanctioned, relative to other societies without such cultural norms. That is, geminophobic cultural norms increased twin mortality rates beyond what would be expected from biological considerations alone, at least until the 1980s (Pison, 1987; Fenske & Wang, 2023). Different groups have developed a number of rationalisations to justify their negative beliefs about twins. Some cultural groups hold that the birth of twins is evidence of multiple paternity and female infidelity (Leroy, 1976; Marroquín & Haight, 2017; Taylor, 1993; Cowlishaw, 1978). Others regard human twinning as an analogue of ‘animal-like’ reproduction (i.e. the rearing of litters), leading some to devalue the humanity of twins, and sometimes their mother too (Leroy, 1976; Marroquín & Haight, 2017). For some groups, twin births even create a dilemma for existing kinship structures and inheritance systems (Turner et al., 2017), a dilemma that can be ‘resolved’ by legitimising twin infanticide (Marroquín & Haight, 2017). Finally, the arrival of twins may be considered an economic shock for parents, and the complete withdrawal of parental investment in at least one twin might reflect a ‘rational’ parental investment strategy when a population is facing severe resource constraints (Hrdy, 1992; Ball & Hill, 1996; Marroquín & Haight, 2017). Even in the absence of infanticide, the birth of twins may bear negative symbolic or spiritual connotations, such that parents have to go through purification rituals (Leroy, 1976).

Twinship, however, is also celebrated in many cultures; twins can be a major source of pride and social standing for their parents, and are sometimes even the subject of community-based or kin-based worship. In such geminophilous cultures – the vast majority of which seem to cluster in Sub-Saharan Africa (see Fig. 1b) – several positive and beneficial attributes are associated with twins and their families. Parents of twins frequently take on honourary names that signal their ‘twinner’ status to the community – e.g. in southern Benin and among some ethnic groups in Uganda (Basoga and Baganda; Ayari-De Souza, 2020; Kabagenyi et al., 2016). Among the Kejom, a Bantu group from Cameroon, twinship is traditionally viewed as a way to increase a family's social prestige: twins who are female are introduced to the royal family, potentially to become wives, while twins who are male may be sent to become part of the staff serving the royal family as palace retainers (Diduk, 2001). A number of Sub-Saharan African societies associate twinship with fecundity and regard twins as a source of wealth and good luck for their parents (Leroy, 1976; Schapera, 1927). For example, mothers of twins enjoy special social status among the Lele people of the Democratic Republic of Congo, and both mothers and fathers of twins are thought to have been selected by the spirits to acquire ‘twin magic’ powers that can be used to boost fecundity and good hunting (Douglas, 1957). Similarly, Nigerian Yoruba think that twins bestow wealth and fecundity upon their parents (Oruene, 1985). Mirroring what is found with twin infanticide in geminophobic societies, geminophilous cultural norms may too have detectable demographic or economic consequences – e.g. by leading to better social provisioning of the families of twins, and thus minimising the mortality rates of twins relative to twinship-neutral groups. However, empirical studies on the topic are exceedingly rare. One study in Benin – a country where geminophilous cultures are predominant (Saulnier, 2009) – found that being a twin child is a statistically significant predictor of receiving childhood vaccinations (Budu et al., 2023). This finding replicates a previous study, also from Benin, which presented qualitative evidence that twins are often the recipients of money transfers from unrelated (i.e. non-kin) individuals in their communities (Alidou, 2021).

Social scientists have proposed several evolutionary, social and economic explanations for the development of geminophobic institutions and norms, especially socially sanctioned twin infanticide (Marroquín & Haight, 2017). Comparable explanations for the rise and persistence of geminophilous systems, however, are still lacking, and key theoretical questions remain unanswered. Why should individuals allocate economic resources, or any other currency – i.e. time, energy or prestige – to unrelated individuals, just because they belong to a seemingly arbitrary biosocial category (i.e. that of *twins*)? What is the role played by the supernatural features that are often attributed to twins in maintaining those behaviours? We think a cultural evolutionary perspective provides a unified framework that might help to explain both geographic variation in geminophilous vs. geminophobic norms, and variation in the underlying rates of DZ twinning.

### Explaining between-population variation

The vast majority of studies on twinning take ethnic background to be a relevant risk factor for twinning propensity, but omit any meditations on the causal processes that might have produced such between-group differences. Indeed, the wide diversity in twinning rates between human populations has not, in our opinion, received a satisfactory explanation, and remains a largely under-theorised research area. Early work by Bulmer (1970) on DZ twinning clustered ethnic groups according to their twinning rates, and found that differences between groups were substantial, approaching a ratio of 4-to-1 between the highest (Sub-Saharan Africa) and the lowest (East Asia) values. More recent research has generally confirmed these early findings (Hoekstra et al., 2008b; Smits & Monden, 2011). The scholarly work on twinning in Sub-Saharan Africa has historically focused on the Yoruba – an ethnic group inhabiting southwestern Nigeria and adjacent Benin – which is thought to have the highest twinning rate in the world (Creinin & Keith, 1989). Later demographic research has highlighted how there is a large ‘high twinning’ geographic region in the African continent, stretching roughly from West Africa to Central Africa, with the highest incidence observed in Benin (Smits & Monden, 2011).

As previously outlined, the dominant theoretical explanation for the existence of such a high-twinning cluster in West Africa invokes the localised consumption of yams that enhance twinning rates – presumably because such yams contain estrogen-like substances that boost ovulation rates (Nylander, 1979; Steinman, 2006a). This explanation is corroborated by the observation that twinning rates are higher among women of ‘lower’ social class in Nigeria, who reportedly have a higher intake of yam in their diets, compared with women in the ‘upper’ class who have more ‘European-style’ dietary habits (Nylander, 1978, 1981). Interestingly, locals of the exceptionally high-twinning Nigerian town of Igbo-ora locals do not consider yams to be a causal factor in twin births; beyond factors such as ‘the will of God’ and heredity, residents attribute twinning to the consumption of certain foods containing okra leaves and cassava, but not yams (Omonkhua et al., 2020). As mentioned previously, the ‘local dietary habits’ explanation for high twinning rates in West Africa is, in our opinion, made less plausible by the observations that African-Americans (a population with large West African ancestry) experience higher DZ twinning rates than any other ethnic group in the USA (Khoury & Erickson, 1983; Abel & Kruger, 2012), and that Haiti – a country where 95% of the population is of African descent (Minority Rights Group, 2020) – has the highest DZ twinning rate among all Central and South American countries analysed by Smits and Monden (2011).

Formalising a brief musing from Pison (1987), we propose that population-level diversity in twinning may be attributed, in part, to gene–culture coevolution. More specifically, we suggest that geminophilous cultural norms might be sufficient to either: (1) buffer the resource constraints that lead to higher fitness costs for twin-births; or (2) offset the direct costs of twining by increasing the mate value of twins. Either form of cultural driven selection could, in theory, lead to appreciable changes in the frequency of alleles responsible for regulating ovulation rate, and thus influence the risk of twinning. This idea has not received much prior attention in the literature, and so we dedicate the final section of this paper to sketching a formal version of the argument, deriving empirical implications from the model, and proposing empirical investigations that could substantiate or contradict our central hypothesis.

We remark that we are *not* proposing a univocal, directed causal arrow from culture to genes, but rather a dynamic process, where both factors influence each other. Natural variation in twinning rate between groups – possibly owing to the ecological circumstances highlighted in Box 1 – might trigger diverse cultural responses in different places, which in turn impact gene frequency. We grant that it is tempting to simply attribute the high salience of twinship in Sub-Saharan Africa to the high twinning rates found in the region – rather than consider cultural practices and genetic variation as a dynamically linked system. In fact, it is rather normative to think of culture as being ‘without teeth’ – and only responding to genetic and ecological factors (Harris, 2001), rather than causing changes in such factors. However, in recent years, the ability of culture to profoundly shape the natural environment at both local scales – though paradigms like *niche construction* (Laland et al., 2001) – and global scales – through investigation of *human dimensions of climate change* (Gibson et al., 2000) – has come into sharper focus; culture has teeth.

The science of gene–culture coevolution is still fairly new, and robustly verified empirical examples of gene–culture coevolution are still rare. However, the framework has been applied to study phenotypes as diverse as lactase persistence (Beja-Pereira et al., 2003), human handedness (Laland et al., 1995), culturally driven sexual selection (Laland, 2008), primary sex ratios (Kumm et al., 1994; Kumm & Feldman, 1997), malaria resistance (Laland, 2008), and the relationship between cannibalism and selection on genetic variants conferring resistance to prion-disease pathology (Mead et al., 2003; Collinge et al., 2006). In such cases, the emergence of genetic adaptations is proposed to be a direct consequence of cultural behaviours (e.g. cannibalistic mortuary feasts) or technological achievements (e.g. animal domestication). Because cultural systems can create arbitrarily strong selection gradients, genetic responses to cultural content can be rapid and strong.

In the case of the cannibalistic ‘transumption’ documented in New Guinea, mortuary feasts in which human brain tissue was consumed spread a slow-acting, but invariably fatal prion-disease (kuru) widely, and within a period of decades, kuru became the most common cause of death of women in affected villages (Collinge et al., 2006). Because heterozygotes for the PrP glycoprotein were less susceptible to infection and disease progression, between-population differences in allele frequency for PrP were detected after a relatively short period of time (see Ross & Richerson, 2014, for additional comentary). More recent work has even discovered directional selection on a genetic variant – PrP G127V – that confers resistance to prion disease; this allele was found to be present only in individuals living in the geographic region where kuru was common – not in unexposed population groups worldwide – and it was not found in patients experiencing the symptoms of kuru (Mead et al., 2009). Although we do not expect effects nearly as strong in the case of twinning rate, the cultural institutions influencing twinning rate are arguably more enduring, and should be expected to have smaller effects integrated over longer periods of time.

### Twinship beliefs as cultural adaptations

Humans are a unique species and have an unprecedented capacity to devise elaborate cultural adaptations (i.e. behavioural adaptations that are socially transmitted) in order to cope with radically different environments (Henrich & McElreath, 2003). For example, historical taboos against the consumption of certain foods limited consumption of dangerous toxins (Henrich & Henrich, 2010), and social and religious norms regulating the use of ecosystems (Lansing, 1987) may prevent groups from overexploiting natural resources (Colding & Folke, 2001). Nevertheless, cultural traits are not always adaptive: cultural evolution can also produce ‘maladaptive’ traits that stably persist owing to the same kinds of social learning biases that spread adaptations (Boyd & Richerson, 1988). For example, harmful practices such as foot-binding and female genital modification/mutilation appear to be maintained by frequency dependence (Mackie, 1996; Ross et al., 2015, 2016). Here, however, we will argue that both geminophobic and geminophilous systems might be understood as cultural adaptations to the challenges of twin-births. We then draw on work in the field of gene–culture coevolution in order to investigate the formal linkages between cultural adaptations and induced selective pressures at the genetic level (Feldman & Laland, 1996).

The diverse ways in which human cultures deal with the concept of twinship are directly related to basic trade-offs regarding reproduction and the survival of twins. The challenges of twin-births are well established in the scientific literature, as outlined earlier. Moreover, there is evidence that, cross-culturally, parents of twins are well aware of the hazards that twin births entail (Pector, 2002). The emergence of geminophobic systems, which hold open hostility towards twins (to the point of permitting infanticide against one twin), can be understood as cultural practices designed to minimise the probability of parents losing both children by spreading maternal resources too thin. This argument for infanticide as a ‘rational’ parental investment strategy has received plenty of attention in the human evolutionary sciences: if divided investment in both twins yields fewer expected surviving offspring than undivided investment in a single twin, then there may be a potentially adaptive rationale for the emergence and persistence of twin infanticide in challenging ecologies (Hausfater, 1984; Hrdy, 1992; Ball & Hill, 1996). An unintuitive consequence of the practice of twin infanticide, however, is that it should actually *reduce* the strength of selection against alleles responsible for polyovulation relative to societies where twin infanticide is socially prohibited, but social support systems for mothers of twins are absent. Because twin infanticide (if adaptive) reduces the fitness burden associated with twin births, it must also decrease the scope for selection to act against twinning propensity.

Another – arguably Pareto improving – approach for reducing the potential costs of twin births draws on the unique ability of humans to organise collective action. In geminophilous systems, mothers of twins might not have to choose between investing in a particular twin, but may instead reach out to their communities in order to acquire sufficient resources to raise both. In other words, geminophilous rituals, beliefs and behaviours may have emerged as an insurance system aimed at offsetting the hazards of twinning. Individuals may be willing to allocate resources to non-kin twins in their community, with the expectation that they too will be the recipients of communal resources if and when twinning should occur to them. Such cultural institutions are not simply vague possibilities. During ethnographic research in Benin, we observed several women moving through the villages, imploring others in the community to share a small amount of money with them – these women not only brought their twin children with them and referenced their special needs when soliciting support, but they also carried statues representing deceased twins in their families, making the consequences of withholding support clear in the minds of those they interacted with.

Such a mechanism is more likely to develop in contexts where individuals have a reasonably high expectation of having twins at some point in their lives – e.g. in high-fertility regimes (more details in Box 2). Until the demographic transition, most agrarian societies – including those in which many geminophilous systems developed – were characterised by such demographic regimes. Faced with an appreciable risk of giving birth to twins at some point in their lives, individuals might be incentivised to create institutions designed to offset the costs of twinning. Such cultural institutions may provide socially regulated means of obtaining material support (e.g. food, childcare and other forms of aid) for twins and their families. When twinning is not particularly common, the *per capita* costs of providing such insurance can be quite low, and still produce substantial benefits for rare twinners. In Box 3, we provide an initial framework to model such a system, and show that once geminophilous norms are common, selection will favour genetic variants that increase twinning propensity.

Box 2: Prevalence of mothers-of-twins in different fertility regimes

A probably underappreciated idea is how high the prevalence of mothers-of-twins may be in societies with ‘natural fertility’ regimes. A substantial frequency of mothers-of-twins may have implications for the development of cultural norms designed to support such women. The probability of ever becoming a mother-of-twins, *T*, conditional on a constant probability of twinning, *x*, per pregnancy, and a number of successful pregnancies, *P*, is given by the following equation:6
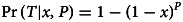

This equation assumes that the number of pregnancies, *P*, is independent of twinning (i.e. twinning does not reduce the number of future pregnancies), and that *x* remains constant across age and parity. With *P* = 2 and a 1.3% twinning probability (*x*_*l*_ = 0.013), the proportion of twinning women is ~2.6%. *P* = 2 reflects the demographic pattern in developed economies, while 1.3% reflects the current global average twinning rate. In contrast, in a pre-demographic-transition, high-fertility context, which better reflects the demographic regimes where geminophilous norms and beliefs developed, *P* may be as high as ~10. A twinning probability of *x*_*h*_ = 0.045 (e.g. as observed among the Yoruba) and a fertility of *P* = 10 would lead to ~37% of women being mothers of twins at some point in their lives! If we plug the standardised twinning rate (*x* = 0.028) and the completed fertility rate (*P* = 5) of modern Benin – the current highest-twinning country – into Eqn (6), we estimate that ~13.2% of women end their reproductive careers as mothers of twins.

**Figure 3.**
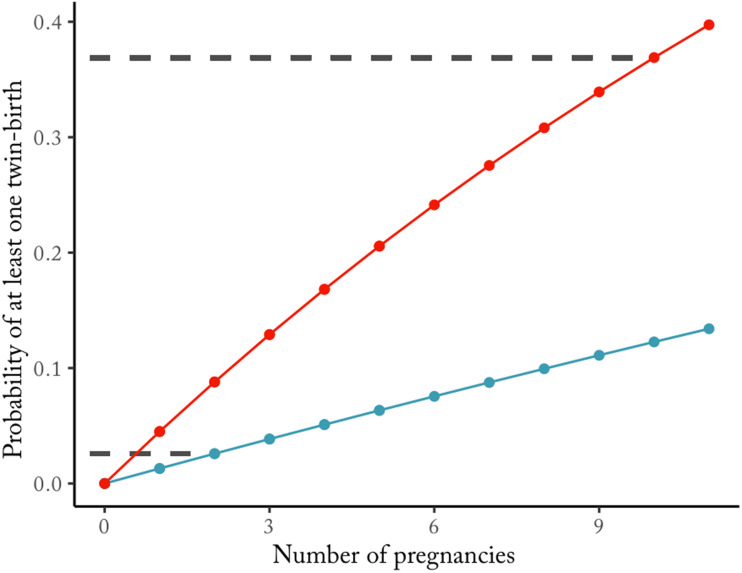
Cumulative probability of at least one twin birth, as a function of twinning probability (*x*_*l*_ = 0.013 blue, and *x*_*h*_ = 0.045 red) and number of pregnancies. The horizontal dashed lines show the *y*-axis intercepts of the numerical examples given in the box.

Figure 3 plots the function in Eqn (6) for two values of *x*, reflective of low and high twinning probability. This simple example illustrates how twinning might be something that occurs for a relatively high proportion of parents in a high-fertility context, making its potential cost apparent and salient.

Box 3: A modelling framework for culture-led natural selection

Here, we show that geminophilous cultural systems can exert selective pressure in favour of genotypes for higher twinning propensity, even when twinning is costly.

Let us assume that two genotypes exist, a low-twinning genotype, *Q*, and a higher-twinning mutant, 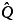. Assume also that two cultural types exist, *G* and *A*. *G* is a cultural variant that produces geminophilous support networks, and *A* is a cultural variant that does not produce such support networks. Four types are then possible: *AQ*, 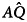, *GQ*, and 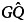.

All individuals have *M* units of material resources and produce one birth per generation. Some percentage, *β*, of births are twin-births for low-twinning *Q* types, and a higher percentage, 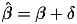, are twin-births for high-twinning 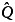 types.

Relative fitness is determined by offspring survival, which is affected by resource investment. Twinning is maladaptive when the expected number of surviving offspring is lower for twin than singleton births. We assume that survival, *S*( ⋅ ), with output on the unit interval, is a smooth and monotonically increasing function with respect to its argument. The expected number of surviving offspring from a singleton birth is simply: *S*(*M*). In a twin birth, each offspring receives a half-share of resources, *M*/2, but the parent has twice as many offspring. The expected number of surviving offspring from a twin birth is thus:
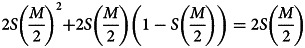

Singleton births will be favoured by selection when the following functional inequality is satisfied:7
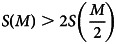

We assume that *S* and *M* are such that Eqn (7) holds in the absence of any special institutions to buffer the costs of twinning.

A exponential cumulative distribution function of the form: 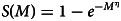, can be used to parameterise the offspring survival function in numerical implementations of our model.

The expected fitness of non-geminophilous, low-twinning individuals is:8
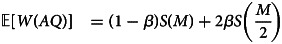

The expected fitness of 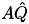 individuals is similar, but with *β* being replaced by 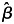, leading to higher production of twins. When Eqn (7) holds, 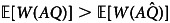, and so the twinning rate should never increase when the population is purely of type *A*.

Next, we consider the fitness of geminophilous, low-twinning individuals, *GQ*. All *G*-type individuals pay costs for maintaining the geminophilous system. There is a fixed cost, *γ*, to be a member of the support network, and a variable cost, *α*(*M* − *γ*), that is paid to the community pool by individuals who *do not* produce twins. Individuals who *do* produce twins, instead receive a payout from the community pool. For a *GQ*-type individual, at each generation, we assume that a twin birth is a Bernoulli random variable, with probability *β* (and similarly for 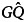 types with probability 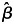).

The total pool of resources available to be shared by all individuals who produce twins is a random variable *κ*, defined later. Each individual who produces twins gets an equal share of the pool, so *κ* is scaled by 1/*T_G_*, where: 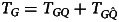 is a random variable giving the total number of twin births of geminophilous individuals. Thus, at any generation, the resources available to be split among the twins of a given parent is:
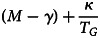

And so, the fitness expression is:9
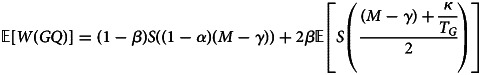

As before, the fitness of the 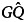 type follows the same form as the above, but with *β* being replaced by 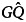

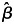
.

To interpret Eqn (9), we need to describe the redistribution mechanism. In total, the redistribution pool, *κ*, will have *α*(*M* − *γ*) units of resources, per *G*-type individual *that did not produce twins*, as all *G*-type individuals producing singletons contribute equally. Letting *N*_*GQ*_ and 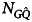 be the number of individuals of type *GQ* and 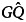, then:10
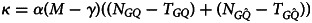

To see if geminophilous cultural systems can favour genotypes for higher twinning propensity, we can assume that the population is composed purely of geminophilous individuals (i.e. 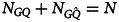), and further that twinning propensity is quite low among individuals of type *Q* (i.e. *β* = 0). Then, we can ask if selection can favour a higher twinning rate – i.e. can 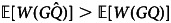
? Even with the above assumptions, however, calculating the expectations is challenging, so we will resolve ourselves to an easier problem for now.

We will consider a simple invasion condition where: *N*_*GQ*_ = *N* − 1, 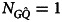, and the lone 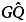-type happens to twin, 
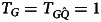
. We first write the fitness expressions for

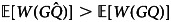

conditional on the above assumptions, and reduce them to yield:11
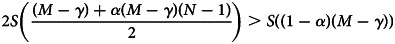

Inequality (11) always holds if the argument of *S* on the left-hand side exceeds the argument of *S* on the right-hand side, since *S* is monotonic. So, to make progress towards an analytical solution, we can solve for a conservative value of *α* sufficient to allow culture-driven selection to favour increases in genetic twinning propensity using the following expression:12
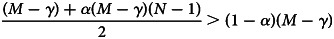

Inequality (12) reduces to the simple condition:
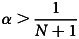

So, if *G*-type individuals have a simple cultural norm such as: *α* ≈ 1/*N_G_* – i.e. in a community of 100 twin-supporters, everyone gives about 1% of their wealth to the redistribution mechanism – then this inequality will be easily satisfied. Even smaller values of *α* will typically be sufficient for inequality (11) to hold. As such, a mutant 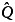 can invade, and culture-driven selection can favour increases in twinning propensity, *δ*, until an equilibrium is reached.

Here, we have only established the simplest of conditions: that – once the *G*-type is common – culture-driven selection can increase twinning frequency by decreasing offspring mortality. We leave a fuller description of the invasion and stability conditions, as well as numerical simulations of the full stochastic model, to a more technical model paper.

An additional mechanism by which geminophilous norms might increase the frequency of twinning relates to the conferral of prestige and enhanced social status on twins and/or their parents, as such social standing may have consequences for reproductive success (Redhead & Power, 2022). A positive association between twinship and prestige/social standing in some geminophilous societies is qualitatively reported in the ethnographic literature (e.g. Diduk, 2001), and social status has been found to be a predictor of reproductive success in many non-industrialised societies, at least for males (Von Rueden & Jaeggi, 2016; Ross et al., 2018, 2023). Although we know of no data on the differential fertility of twins vs. singletons in geminophilous societies, this mechanism is plausible.

A recurrent feature of both types of twin-related cultural systems is the association of twins with supernatural characteristics. Such attributes do not have to be clearly positive or negative, but may be ambiguous. Indeed, in some cultures, twins are believed to possess supernatural powers that can be variably used in a destructive or in a beneficial way, depending on whether they are well cared for by kin and community members (Leroy, 1976). The ascription of supernatural features to twinship could be understood as a tool to increase compliance to twin-related cultural norms, be they geminophilous or geminophobic. Work in the evolutionary anthropology of religion suggests that several religious prescriptions may have developed to effectively enforce prosocial and cooperative norms via the threat of supernatural punishment (Fitouchi et al., 2023). Delegating norm enforcement to supernatural forces may constitute a convenient and efficient way to circumvent the problems stemming from punishment and monitoring costs, and thus extend support networks beyond immediate kin (Purzycki et al., 2016).

### Testable implications

There are several testable implications of our key ideas. First, twinning rate – and possibly genetic variants influencing polyovulation – should spatially covary with the distribution of geminophilous and geminophobic norms. Second, the survival of twins should be higher in geminophilous societies compared with twinship-neutral (and, obviously, geminophobic) societies in comparable environments, as geminophilous institutions are only effective if they substantially reduce the costs associated with twinning. Third, in geminophilous societies, twinship should be associated with elevated reproductive rates, both because geminophilous institutions buffer the cost of twinning, and because increased prestige should confer social advantages (e.g. in the mating market). Finally, we predict that a cultural phenomenon called ‘twinship hijacking’ should only be found in geminophilous societies. In the rest of the subsection, we articulate these implications.

To address the first implication, spatial regression analyses can be used to test for statistical associations between twinning incidence and the presence/strength of twin-related cultural institutions. We present a preliminary synthesis of such spatial data in Fig. 1, by merging a cultural dataset from Fenske and Wang (2023) with a twinning-rate dataset from Smits and Monden (2011). The evidence is suggestive of a possible positive association between the presence of geminophilous norms and the incidence of twinning, but the coarse-grained (i.e. country-level) nature of these data is not optimal. Ideally, both anthropological (i.e. twin-related norms) and epidemiological (i.e. twinning rates) information should be aggregated at the smallest geographic unit possible, and analysed with robust tools (e.g. regression discontinuity designs: Keele & Titiunik, 2015).

Some geographically resolved studies on twinning have already been conducted. For example, country-level twinning rates in the developing world have been produced (Smits & Monden, 2011), and the impact of twinship beliefs on twin mortality in Sub-Saharan Africa has been investigated (Fenske & Wang, 2023). Concordant with our expectations, twins in historically geminophobic societies experienced disproportionately high mortality rates compared with twins in non-geminophobic societies until the 1980s, especially in rural areas (Fenske & Wang, 2023). As previously mentioned, there is also contemporary evidence that twins are more likely than singletons to receive childhood vaccinations in geminophilous Benin (Budu et al., 2023). Direct evidence that geminophilous norms incentivise preferential investment into twins, reducing their mortality disadvantage, may affirm our hypothesis.

Comparable analyses have been used to test for associations between twinning propensity and fertility outcomes, mostly using data from pre-industrial Europe (Rickard et al., 2022; Lummaa et al., 1998; Gabler & Voland, 1994; Haukioja et al., 1989). With the exception of Sear et al. (2001), no such analyses have focused on Sub-Saharan Africa, where twinship is especially salient in a wide variety of ways. Furthermore, most studies focus on whether the *twinning propensity* of women is associated with completed fertility and/or other relevant life history traits (i.e. body mass, or age at first birth), while generally overlooking the fertility outcomes of *twins* themselves (but see Gabler & Voland, 1994, for an exception). Additionally, spatially resolved, genomic data are becoming increasingly available (e.g. Smetana & Brož, 2022), and may permit studies exploring geographic structure in the distribution of candidate genes for human polyovulation.

Finally, we expect *twinship hijacking* to occur only in geminophilous societies. We use the term twinship hijacking to refer to a phenomenon whereby individuals attempt to expand the social concept of twinship beyond mere biological twinship, and in doing so manipulate the symbolic system in order to reap the benefits associated with twinship. An example of twinship hijacking – that we have noted through first-hand ethnography in Benin – is that singletons born via breech delivery (i.e. legs-first) are sometimes socially considered as ‘twins’ (see Renne & Bastian, 2001, for similar findings elsewhere). Breech babies, along with twins, are considered as ‘sacred children’ in West-Africa-derived Haitian Voodoo as well (Peek, 2011). Conversely, we expect no such expansion of the social category of ‘twin’ in geminophobic societies, as parents will have no incentive to claim or convince others that their child is a twin in contexts where twinship does not lead to special affordances.

### An empirical challenge: the curious case of the Yoruba

One challenge to our argument involves a potential cultural switch experienced by the Yoruba nation. As mentioned earlier, the Yoruba are among the highest twinning ethnic groups in the world (Creinin & Keith, 1989), and some evidence suggests that the birth of Yoruba twins was historically regarded as an ominous event, and that twin infanticide was practised (Chappel, 1974). The historical negative valence of twin births apparently stemmed from the belief that twin births were a consequence of adultery (Hall, 1928; Chappel, 1974; Oruene, 1983). If this evidence is true, it would problematise our argument of geminophilia exerting selective pressures in favour of twinning propensity in West Africa. Presently, Yoruba culture is highly geminophilous, but if such cultural practices are relatively new, it is unlikely that there would have been enough time for such cultural practices to increase twinning rate via selection.

The existence and timing of such a cultural reversal, however, is extremely unclear, as are the causes, because most available information is based on oral histories. Qualitative interviews in Nigeria produced very little consensus among local interviewees about the reasons for the cultural change, and even when it occurred (Chappel, 1974). There is, however, evidence of appreciable spatial structure in the degree of geminophilous vs. geminophobic norms among Yoruba historically (Renne, 2001). This implies that, perhaps, there was no major historical switch affecting all Yoruba, but rather, that different sub-populations settled into different cultural equilibria.

If the Yoruba system switch is both: (1) true (i.e. a system reversal actually did take place starting from baseline geminophobic attitudes); and (2) relatively recent (the past 200 years or so), it would indeed be harder to take the high twinning rates of some West-African ancestry populations as evidence of selection driven by geminophilous cultural attitudes. However, given that the Yoruba population represented a substantial proportion of the enslaved Africans brought to New World colonies (Hall, 2005; Zakharia et al., 2009), and that twins are worshipped in Yoruba-influenced syncretic religions across the American continent – including Candomblé in Brazil and Santeria in Cuba (Leroy et al., 2002) – we are suspicious that geminophobic norms were common across all Yoruba populations immediately prior to the Atlantic slave trade. This would also raise an interesting question in cultural anthropology as to why several Yoruba-descending groups in the New World hold geminophilous attitudes if the originating culture at the time of the slave trade was explicitly geminophobic and practising twin infanticide.

## Conclusion

The sizeable variation in twinning rates observed between human populations still lacks a satisfactory scientific answer. To address this open problem, we have formulated an account of such diversity that incorporates both demographic observations and qualitative ethnography within a gene–culture co-evolutionary framework. We envision two main avenues of research to further develop and test this hypothesis. *In primis*, our arguments about the evolution of twinship institutions and their impact on genetic propensities for polyovulation need to be translated into a complete evolutionary model, in order to test their internal validity. The model that we put forward here is an initial step towards this goal. *In secundis*, empirical evidence from fine-grained ethnographic and demographic data in rural populations would be highly valuable.

Anthropologists and demographers working with communities where twinship is salient might design questionnaires to: (1) identify twinship norms; and (2) collect information on different aspects of social and economic life which may impact survival and fertility outcomes. Information on wealth and income, social status and prestige, and social network structure would then permit tests for causal paths linking twinship institutions and survival and fertility outcomes. Statistical analyses informed by causal reasoning would be necessary to disentangle the multiple phenotypic confounds that similar studies conducted in the past have encountered.

The ethnographic approach, however, is not without limitations – most importantly, globalisation, medical and technological advances, and demographic transition are rapidly changing the landscape of human reproductive behaviour. Traditional attitudes towards high fertility are declining worldwide, access to modern healthcare is improving child survival outcomes and exposure to international media is changing many traditional cultural institutions. In sum, the effect of traditional cultural institutions on demographic outcomes in contemporary times may be softened or even null – especially in urban populations worldwide. Indeed, in present-day Sub-Saharan Africa, twin mortality does not covary with past history of twin infanticide anymore (even though it did 40 years ago; Fenske & Wang, 2023), and twinship salience may be declining owing to the impact of Western norms and the greater role played by Abrahamic religions (Renne, 2001). Therefore, while evidence of twinship positively impacting contemporary fitness outcomes would *potentially* corroborate our central hypothesis, null results would not necessarily invalidate it.

In contrast to the ethnographic approach, methodologies linking contemporary genetic variation to historical cultural institutions are less sensitive to the changes brought by modernisation. Because genetic change is typically slower than cultural change, we should be able to find signatures of past selection on genes related to polyovulation even if recent cultural changes have weakened the effectiveness and salience of twinship beliefs and institutions. Using European ancestry cohorts, Mbarek et al. (2024) found signatures of past selection against some alleles associated with DZ twinning; if their analyses are applied to data from individuals of African ancestry, our theory would predict signatures of positive or balancing selection on at least some alleles associated with DZ twinning rate.

In sum, we believe that the lens of gene–culture co-evolution may help to rigorously explain group-level diversity in human dizygotic twinning. Such an approach will require both mathematical modelling and empirical data. We hope that our perspective will encourage future scholars to tackle the enduring evolutionary puzzle of human twinship.

## Data Availability

No new data were created or analysed for the main conclusions in this study. However, Figure 1 in the manuscript contains a preliminary statistical analysis using already published data, including an index of geminophilia for African countries which we have constructed from figures in Fenske and Wang (2023).

## Research transparency and reproducibility

All supporting files and scripts used in our research can be found at: https://github.com/ADR1993/evolutionary-puzzle-twinship.
